# Toxicity of percolates from geotechnical applications with water treatment sludge incorporation

**DOI:** 10.1007/s11356-026-38303-9

**Published:** 2026-10-21

**Authors:** Gabriela de Almeida Nascimento, Suzelei Rodgher, Jorge Kennety Silva Formiga, Fabiana Alves Fiore

**Affiliations:** 1 Department of Environmental and Civil Engineering, São Paulo State University - UNESP, Bauru, SP Brazil; 2 Environmental Engineering Department, São Paulo State University - UNESP, Institute of Science and Technology, São Paulo State University – UNESP, São José dos Campos, São Paulo, Brazil; 3 Environmental Engineering Department, São Paulo State University - UNESP, Institute of Science and Technology, São José dos Campos, São Paulo, Brazil

**Keywords:** Water Treatment Plant Sludge, Sludge dewatering methods, Pilot-scale percolate, Soil-sludge mixture, Algal ecotoxicity assessment, Geotechnical sludge reuse

## Abstract

Although Water Treatment Plant Sludge (WTPS) is viable for geotechnical reuse, its environmental safety regarding realistic percolate generation remains underexplored. This study investigated the ecotoxicity of field-condition percolates using pilot-scale simulations with neat WTPS and a 50% clayey soil-sludge mixture. Over 12 months, tropical rainfall regimes were simulated to evaluate seasonality and two WTPS dewatering pre-treatments: centrifugation and natural drying. Acute ecotoxicity was assessed using the microalgae *Raphidocelis subcapitata*. Results revealed the dewatering method as a critical toxicity determinant: while neat centrifuged sludge percolate caused 94% growth inhibition, its 50% incorporation into the clayey soil matrix reduced this to 3%. Conversely, naturally dried sludge and its 50% mixture maintained high toxicity (>72% inhibition), with seasonality showing no significant effect. These findings demonstrate that WTPS reuse requires careful environmental management, as toxicity is driven by dewatering methods and soil matrix interactions. Crucially, ecotoxicity testing is not mandatory under Brazilian standard NBR 10004, which classifies WTPS as non-hazardous based solely on physical–chemical parameters. Incorporating ecotoxicological evaluation into waste regulation is therefore essential for safe WTPS integration into circular economy models, as percolate monitoring reveals clear environmental risks.

**Graphical Abstract:**

**Figure:**
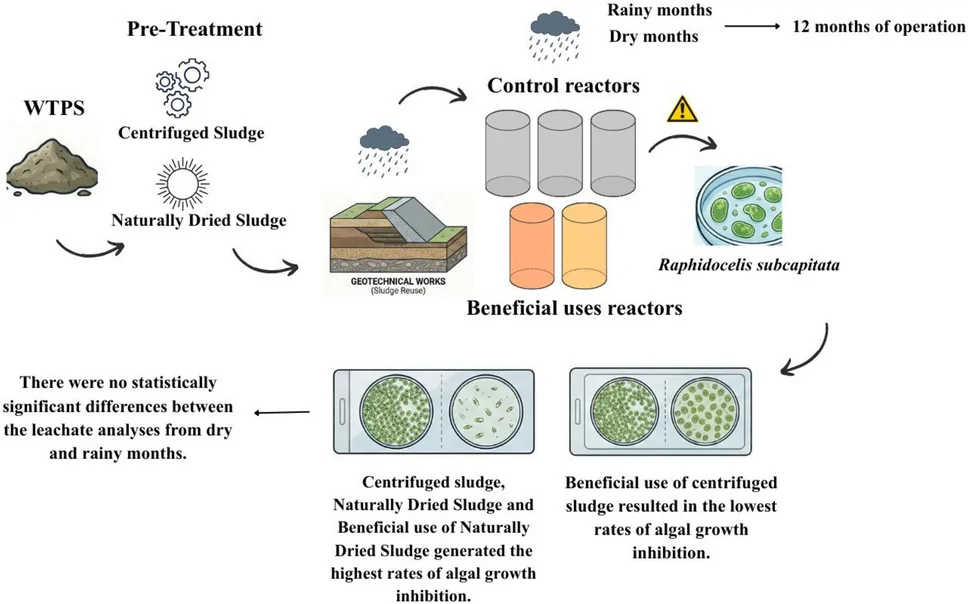

**Supplementary Information:**

The online version contains supplementary material available at https://doi.org/10.1007/s11356-026-38303-9.

## Introduction

Water Treatment Plant Sludge (WTPS) is an inherent waste of water purification (Seckler 2017). Its reuse is widely debated given the high availability driven by global drinking water demand (Nguyen et al. 2022; UN 2025), whereas its inappropriate disposal poses significant ecotoxicological threats to ecosystems (Mateus et al. 2026), further highlighting the necessity for safe re-applications, such as geotechnical reuse.

WTPS is composed primarily of water (~90%) and inorganic compounds originating from raw water and chemicals added during treatment and dewatering (Souza et al. 2024; Messa et al. 2025). Its characteristics vary according to the processing method applied—such as conditioning, centrifugation, or thermal and natural drying (Silva et al. 2025). These processes significantly alter the physical structure and chemical binding within the sludge matrix, directly dictating leachate properties and contaminant mobility (Souza et al. 2024; Messa et al. 2025; Silva et al. 2025).

WTPS have been shown to be a technical alternative in geotechnical applications, such as application in landfill coverage (Boscov et al. 2021), use as a pavement base or sub-base (Fiore et al. 2022), as a raw material to build ceramic products, lightweight aggregates, and concrete (Gomes et al. 2019). However, reuse of WTPS must unite technical and economic advantages. As highlighted by Urban (2017), the valorization of WTPS should not be a lower-cost alternative but a sustainable practice, necessitating a deeper understanding of its long-term environmental impacts and ecotoxicological safety.

To ensure safe and sustainable reuse practices, analyzing the toxicity of WTPS is necessary (Almeida et al. 2025), as potential contaminants present in the WTPS can be carried to other environmental compartments (Acquolini 2017). The environmental toxicity of chemical agents is a widely discussed topic due to their negative impacts on ecosystems and human health (Mustafa et al. 2024; Mateus et al. 2026), such as biodiversity loss and bioaccumulation of toxic substances (Badawi & Madgy 2023).

The first studies related to the toxicity of WTPS were initiated in the 1990 s with freshwater algae, protozoa, fish, and marine bacteria (George et al. 1995). In general, most studies discussing the ecotoxicity of WTPS for aquatic species are carried out with crustaceans, followed by bacteria, microalgae, algae, and fish (Bernegossi et al. 2022). Among these organisms, microalgae stand out for their high sensitivity to chemicals, for being at the base of the food chain, and participating in the processes of energy flow and biogeochemical cycles in aquatic environments (Chapman, 2013).

In the aforementioned studies, the toxicity of WTPS was associated with the presence of metals in its composition, supporting the importance of toxicological tests (Messa et al. 2025). In sludge from WTPs that use aluminum coagulants, Al, Ba, Fe, Zn, Cu, Cd, Ni, Pb, and As have already been found (Cardoso et al. 2019; Hidalgo et al. 2017). Among the aquatic organisms evaluated by Bernegossi et al. (2022) in relation to WTPS toxicity, algae were the most sensitive, since this toxicity can generate inhibition in algal growth, which can reduce the amount of food available for higher trophic levels and negatively influencing water quality, as the imbalance between photosynthesis and respiration reduces oxygen production and pollution control (George et al. 1995).

The microalgae *Raphidocelis subcapitata* is frequently used as an organism-test in toxicity tests (Brazilian Nation Standards Organization—ABNT 2023; OECD 2011; USEPA 2012). Regarding the ecotoxicological evaluation of WTPS, it is noted that leached extracts are used as samples in toxicity tests (Fiore et al. 2022; Agarwal & Singh 2023), which can be explained by the normative guidelines currently in Brazil (ABNT 2004). However, this practice does not represent the real conditions of application of WTPS in soils subject to weathering and can influence the final result obtained (Rodgher et al. 2023).

Previous studies also have demonstrated that sensitivity of *R. subcapitata* in responding to toxicants and effluents was very similar or higher compared to other representative species from different taxonomic groups (Vilas–Boas et al. 2020; Venâncio et al. 2023; Machado et al. 2025; Albuquerque et al. 2026; Zhang et al. 2026). In addition, microalgae are the base of water food chain; their sensitivity suggests that chemical discharge, even partly diluted, may alter primary productivity, animal grazing, nutrient cycling, and energy flow, potentially propagating indirect effects to higher trophic level in food chain (Nie et al. 2013).

Advances in science have contributed to the development of legislation aimed at the proper classification, control, and disposal of potentially hazardous waste (Messa et al. 2025). In the European Union and the United States, guidelines establish criteria for waste classification that consider toxic effects on human health and the environment (European Union 2014; Epa 1976). In Brazil, the Regulatory Standard (NBR) 10,004 classifies WTPS as Non-Hazardous and Non-Inert Waste and exempts the need for toxicity testing (ABNT 2024a, b, c).

Despite its importance, the potential ecotoxicological effects of WTPS reuse in geotechnical works are little discussed in the literature (Souza et al. 2024). When applied in geotechnical works, the primary environmental hazard is the vertical percolation of rainwater through the soil-sludge matrix, which can transport solubilized trace elements into underlying groundwater reserves or surface water runoff (Kanmani & Gandhimathi 2013; Xian et al. 2024). Furthermore, studies evaluating the direct toxicity of actual WTPS percolates—the real effluent that comes into contact with the environment—remain scarce. Although standardized leaching protocols are widely applied, evaluating the direct toxicity of liquid fractions from WTPS has gained increasing attention to better represent potential environmental risks (Jabłońska-Trypuć et al. 2020).

Therefore, the objective of this research was to evaluate the toxicity of samples from field-simulated WTPS reuse conditions in geotechnical works, using the microalgae *R. subcapitat*a as a test organism. We hypothesize that field-simulated percolates differ in toxicity from laboratory extracts because static leaching tests rely on aggressive batch agitation and high liquid-to-solid ratios, whereas pilot-scale percolation involves progressive element wash-out, extended contact times, and chemical attenuation within the soil-sludge matrix (George et al. 1995; Hillel 1998; Rodgher et al. 2023). The results provide evidence to support the safe reuse of WTPS and contribute to the Sustainable Development Goals (SDGs): Clean Water and Sanitation (SDG 6), Sustainable Cities and Communities (SDG 11), and Life below Water (SDG 14) (UN 2025).

## Methodology

**Fig. 1:**
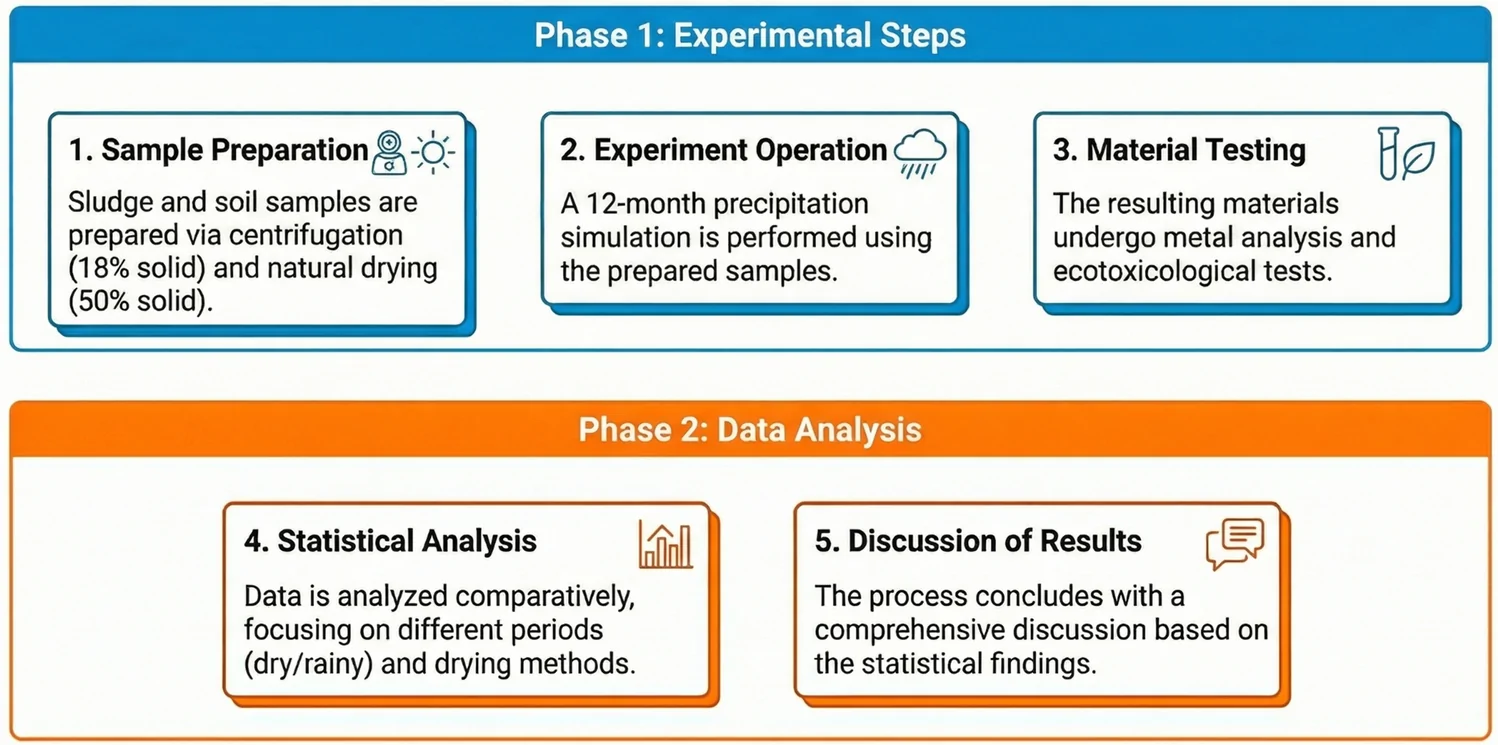
Flowchart of the experimental development and data analysis stages

The experimental methodology (Marconi & Lakatos 2022) was applied in this research, and the flowchart of the development of the stages is shown in Fig. 1.

### Sample collection and experiment preparation

The sludge used in the experiment was collected from a conventional Water Treatment Plant (WTP) on a typical operating day. This WTP is located in the São Paulo Metropolitan Region (Brazil) and uses aluminum sulfate and a cationic polymer as coagulants.

Sludge from the clarification and filter backwash stages is initially conveyed to an equalization tank (∼2% solids) and subsequently to a stabilization tank (∼4% solids). Mechanical dewatering is performed using a decanter centrifuge operating under standard municipal plant parameters (2500–3000 RPM, ∼1500–2000 × g, with a hydraulic retention time of 2–5 min), yielding a cake of approximately 18% solids, in alignment with Brazilian engineering guidelines for water treatment plants (ABNT 1992; Richter 2001). To obtain naturally dried sludge (∼50% solids), the centrifuged material undergoes an open-air drying process in a waterproofed bed over 20 days (mean ambient temperature 22 ± 4 °C, relative humidity 70 ± 10%), featuring periodic manual or mechanical turning (Silva et al. 2025). To guarantee characterization representativeness, composite triplicate samples (*n* = 3) were collected immediately following both the centrifugation and natural drying processes.

The soil, predominantly clayey and commonly used in geotechnical applications (Boscov et al. 2021), was received by donation and characterized in accordance with Brazilian Standards. The soil was used in its raw state, without any prior chemical treatment or purification. The preparation was limited to physical processing, consisting of comminuting the clods to ensure homogeneity before mixing with the sludge. Table 1 presents the evaluated parameters, the methods used, as well as the results.

**Table 1:** Soil characterization

Characterization	Reference	Results
Humidity	NBR 6457 (ABNT 2024a, b, c)	4.65%
Organic Matter	NBR 13600 (ABNT 2022)	1.75%
Liquid Limit (LL)	NBR 6459 (ABNT 2016a, b, c)	37.00%
Plastic Limit (PL)	NBR 7180 (ABNT 2016a, b, c)	25.60%
Specific Gravity (SG)	NBR 7181 (ABNT, 2021)	2.90 g/cm^3^

The chemical compositions of the soil and of the sludge were analyzed prior to reactor assembly, while the physicochemical characteristics of the leachate generated in each reactor were analyzed during system operation. The parameters, sources, and analysis methods are shown in Table 2.

**Table 2:** Analytical parameters, methods, and sample matrices (sludge, soil, and leachate) assessed in the study

Parameter	Sludge	Soil	Leachate
Total metalsᵃ			
Aluminum (Al)	X	X	X
Barium (Ba)	X	X	X
Cadmium (Cd)	X	X	X
Chromium (Cr)	X	X	X
Cobalt (Co)	X	X	X
Copper (Cu)	X	X	X
Iron (Fe)	X	X	X
Lead (Pb)	X	X	X
Magnesium (Mg)	—	—	X
Manganese (Mn)	X	X	X
Nickel (Ni)	X	X	X
Zinc (Zn)	X	X	X
Physicochemical parameters			
pHᵇ	—	—	X
Electrical conductivityᶜ	—	—	X

“X” indicates that the parameter was analyzed for the respective matrix; “—” indicates that it was not analyzed

ᵃMethod: 6010D via ICP-OES (Optima 8300™, PerkinElmer); Reference: SW 846 (USEPA 2018)

ᵇMethod: Potentiometric measurement in a 1:2.5 (w/v) solid-to-water suspension after 1 h equilibration (ABNT NBR 9251:^1986^; equivalent to ISO 10390:2021 (ISO, 2021))

ᶜMethod: Measured in a 1:5 (w/v) aqueous extract using a conductivity meter (ASTM D1125-14) (ASTM, 2014)

### Assembly of the experimental apparatus

Five identical cylindrical reactors were built in PVC, measuring 80 cm in height, 30 cm in diameter and 15 mm in thickness, with a layer of natural gravel at the top (2 cm high) and base (5 cm high) of each reactor, with the aim of ensuring: the accommodation of the materials in the same way as they occur in real application conditions; watertightness; the percolation of precipitated water without the formation of preferential paths; and the drainage and individual storage of the percolate in suitable containers.

To determine the amount of WTPS incorporated into the soil, the study by Fiore et al. (2022) was considered, which shows that there is technical feasibility in incorporating up to 50% of WTPS into clayey or sandy soils. Accordingly, a homogeneous blend was prepared by mixing the soil and sludge in the prescribed proportions and compacted manually using a 3 kg roller. The homogenization process was conducted in laboratory metal trays using specialized manual tools, after which the resulting substrate was transferred into the reactors.

**Fig. 2:**
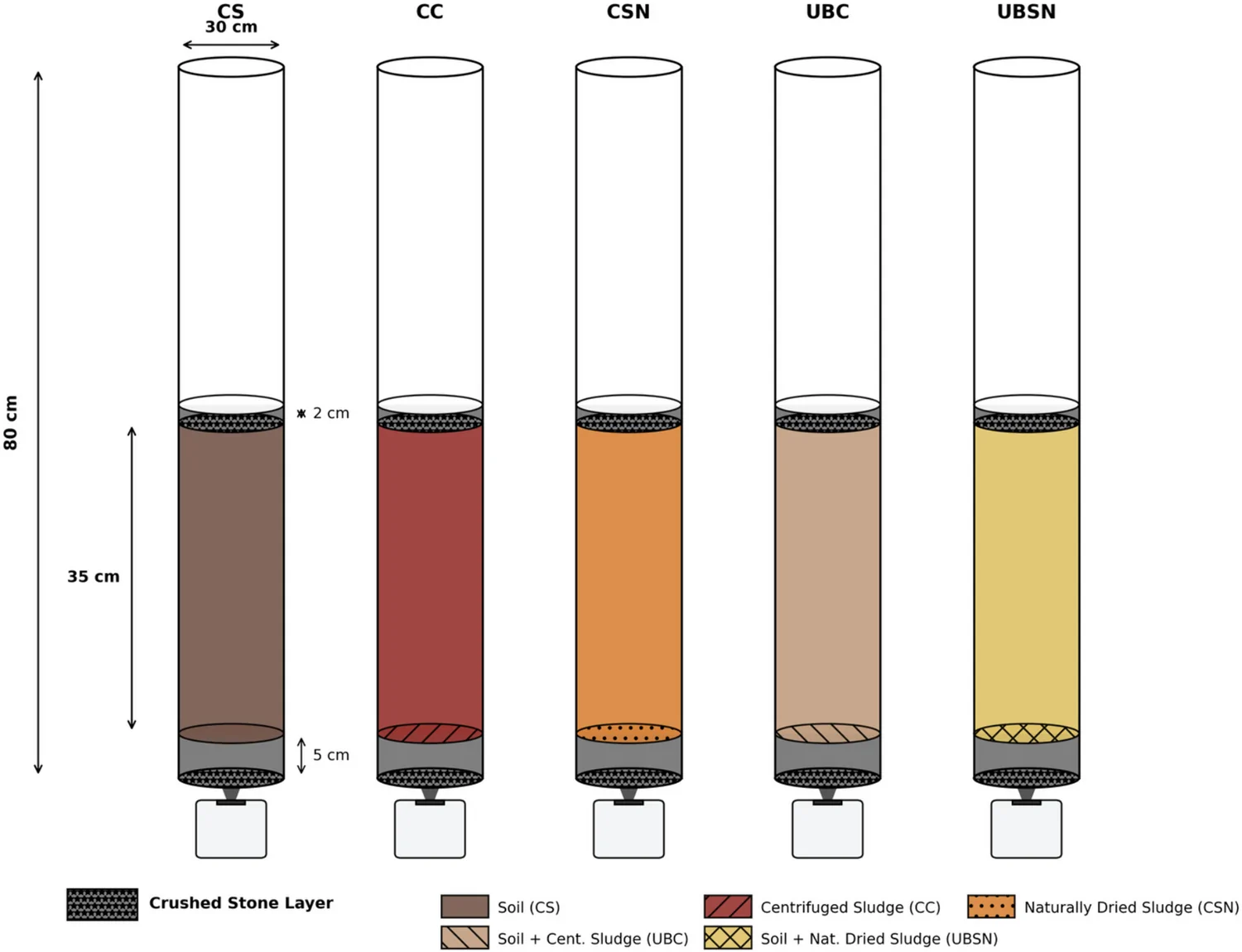
Technical schematic of the experimental setup. Five cylindrical reactors (80 cm height, 30 cm diameter) were mounted on a support structure. Each unit contained a 5 cm base layer of crushed stone, a 35 cm substrate layer (specific to each treatment), and a 2 cm top layer of crushed stone. A collection vessel was positioned at the base of each reactor to receive and store the generated percolate

Due to the physical dimensions, operational complexity, and long-term monitoring required for pilot-scale simulation, each experimental treatment was assembled as a single leaching column (*n* = 1 per treatment condition). Figure 2 shows a schematic representation of the reactors used in the research, and Table 3 shows the nomenclatures used and their respective simulated conditions.

**Table 3:** Simulations evaluated in the study

Reactor identification	Simulated condition
CS	Control—soil (100%)
CC	Control—Centrifuged Sludge (100%)
CSN	Control—Naturally Dried Sludge (100%)
UBC	Beneficial Use—Soil (50%) + Centrifuged Sludge (50%)
UBSN	Beneficial Use Uso—Soil (50%) + Naturally Dried Sludge (50%)

### Experiment operation

The reactors were located in a closed space, and the system operated for 1 year, allowing the simulation of rainy and dry conditions, based on the application of the Water Balance Method (Castilhos et al. 2003). Table 4 shows the volumes of water introduced weekly into each reactor, determined by the historical series of precipitation from a meteorological station installed at the WTP (SABESP 2023).

**Table 4:** Volume of distilled water added to each reactor

Month	Water volume (l)	Month	Water volume(l)
1	3.22	7	0.46
2	2.84	8	0.63
3	2.44	9	1.15
4	1.03	10	1.80
5	1.04	11	1.85
6	0.61	12	2.40

Distilled water was used instead of rainwater to avoid possible interference and maintain system integrity (Almeida et al. 2025). The irrigation occurred manually, allowing the water volume to be distributed homogeneously. Leachate storage containers were changed monthly, and the liquids were preserved with identification, considering the guidelines of the National Guide for Collection and Preservation of Samples (water, sediment, aquatic communities, and liquid effluents) from CETESB (2011).

### Ecotoxicological test

**Fig. 3:**
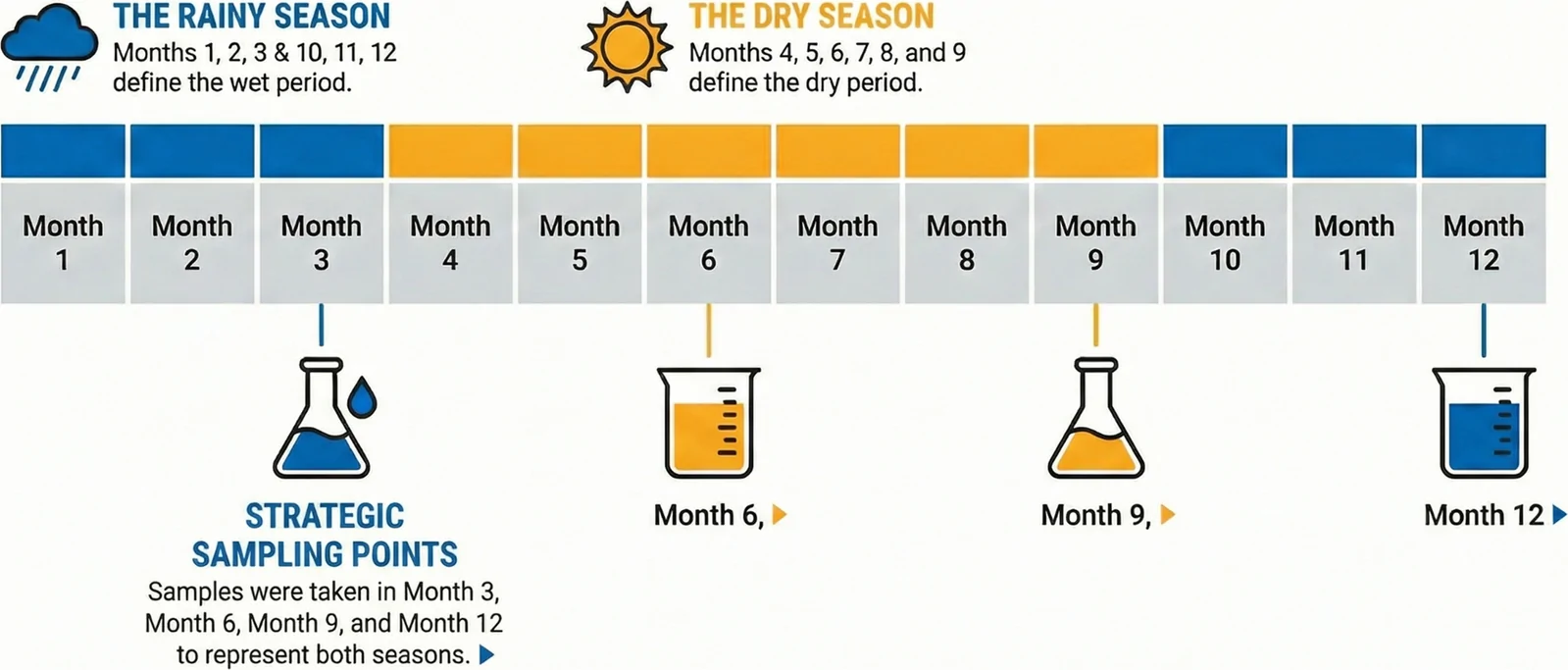
Strategic sampling timeline for ecotoxicological assays with microalgae *R. subcapitata*. The diagram illustrates the division of the 12-month experimental period into the rainy season (months 1, 2, 3, 10, 11, and 12; blue) and the dry season (months 4, 5, 6, 7, 8, and 9; yellow). Sampling points were strategically selected in months 3, 6, 9, and 12 to capture seasonal variability and system stability. This quarterly frequency was defined based on laboratory capacity and the need for representative seasonal data for subsequent metal and toxicity analysis

The number of ecotoxicological assays with the microalgae *R. subcapitata* was constrained to four quarterly samples due to a combination of logistical factors and methodological requirements. This frequency was specifically defined to laboratory capacity and sample preservation constraints, with the need for seasonal representativeness. By conducting four tests over a year, it was possible to capture the distinct variations of the rainy and dry seasons while ensuring the full operational stability of the system, as illustrated in Fig. 3.

The chlorophyte *R. subcapitata* was grown in L.C Oligo culture medium (AFNOR 1980) that does not contain ethylenediaminetetraacetic acid (EDTA). The medium was autoclaved for 20 min at 121 °C in a 250 mL Erlenmeyer flask. The culture remained under illumination (with a 12/12-h photoperiod) of 4524 to 4532 lx, shaking speed of 150 rpm, and temperature controlled at 25 °C ± 2 (ABNT 2023).

Prior to carrying out toxicity tests with samples from the reactors, sensitivity tests with K_2_Cr_2_O_7_ for the microalgae *R. subcapitata* were carried out in order to verify whether the microalgae were suitable for carrying out toxicity tests (ABNT 2023).

In toxicity tests using reactor samples, exponentially growing algal cells were inoculated into each test flask with the 50% diluted sample, in addition to the laboratory control treatment, which contained no sample. A cell density of 1.5 × 10^4^ cells mL^−1^ in exponential growth was added to each test flask. To ensure statistical robustness and reproducibility, experiments were performed in triplicate (*n *= 3) per percolate sample, including control samples, using Erlenmeyer flasks.

*R. subcapitata* cells were exposed to the percolated samples for 72 h; 2 mL aliquots of the toxicity test samples were obtained at the beginning and end of the experiments. All sample collection, test sample preparation, and inoculation with the algal species were performed under aseptic conditions (ABNT 2023). Algal growth in the treatments was assessed by cell counting using an Improved Neubauer-Bright Line hemocytometer at 400× magnification under an optical microscope.

### Statistical analysis

The results were statistically evaluated to determine whether there were statistical differences between the dry and rainy seasons and between the reactors. To assess whether there were significant differences in algal growth generated by the dry and rainy seasons, the Shapiro–Wilk normality test was used on the results from each of the 4 months evaluated. Since the tests did not show a normal distribution, the nonparametric Friedman test was used.

To assess whether there were significant differences between the types of WTPS drying (or between the reactors), the Shapiro–Wilk normality test was used, followed by the nonparametric Kruskal–Wallis test. Multiple comparisons between reactors were performed using the Dwass-Steel-Critchlow-Fligner (DSCF) method. All analyses were performed using Jamovi and OriginLab software.

## Results and discussion

### Analysis of the elemental composition of WTPS and soil

**Fig. 4:**
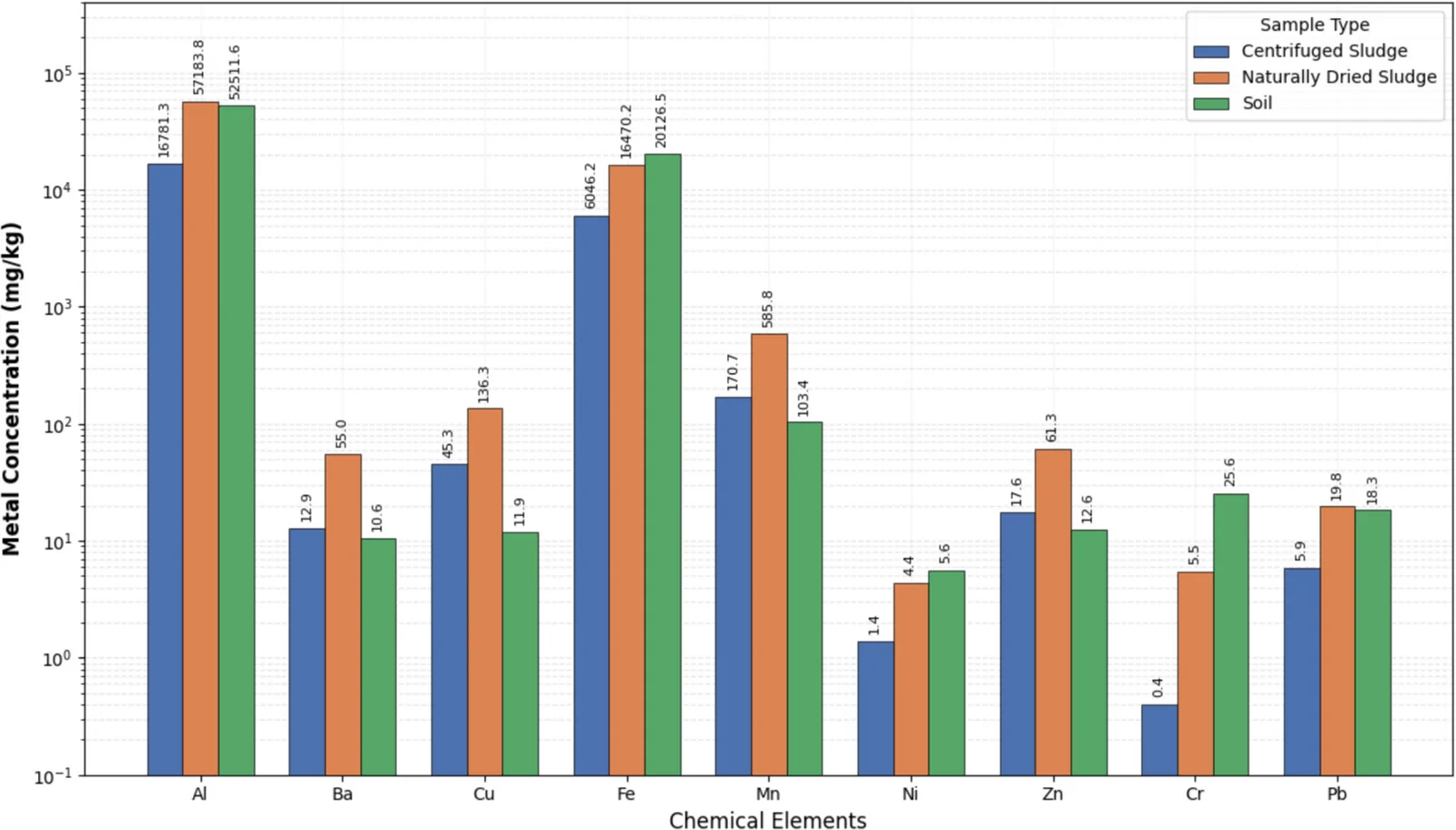
Multi-elemental composition (mg/kg) of soil and WTPS samples. The Y-axis is presented on a logarithmic scale to allow simultaneous visualization of macroelements (Al and Fe) and trace metals (e.g., Cr and Ni). Data represent the initial characterization of Centrifuged Sludge (blue), Naturally Dried Sludge (orange), and Soil (green) used as substrates in the experimental reactors

Figure 4 shows the results of the metal analyses in the solid samples (soil and sludge) used in the reactors.

This result highlights the predominance of Al in the WTPS composition, which may be associated with the type of coagulant used in the WTP. However, the Al concentration in the soil sample is high and comparable to the concentration found in the WTPS, which can be explained by the natural abundance of Al and Fe in Latosols, common in tropical regions (EMBRAPA 2018). It is noteworthy that the metals found in higher concentrations (Al and Fe) do not have limits established in regulations addressing soil contamination (BRASIL 2009; EU, 1986; U.S. Epa 2005).

Although there is regulation that classifies WTPS as non-hazardous waste (ABNT 2024a, b, c), some metals such as Ba, Mn, Ni, Pb and Cr can make the waste hazardous depending on the concentration (ABNT 2024a, b, c; EU, 1986; U.S. Epa 2005), highlighting the importance of prior characterization of the waste and soil before classification and reuse.

### Physicochemical analysis of the leachate

Table 5 shows the results of pH, conductivity, and volume of leachate water in each reactor.

**Table 5:** Results of pH, conductivity, and volume of water percolated in each reactor

	Month	CS	CC	CSN	UBC	UBSN
pH	12	8.02	6.99	7.03	7.01	7.02
	3	7.00	7.99	6.50	6.85	6.45
	6	8.08	8.34	7.47	7.36	7.85
	9	7.77	8.36	7.28	7.17	7.33
Conductivity (μS/cm)	12	71.90	1482.00	1368.00	219.80	1222.00
	3	56.92	1029.00	720.00	78.11	551.00
	6	118.40	964.40	693.70	146.90	591.70
	9	77.51	876.00	557.70	82.41	185.70
Volume of water percolated (L)	12	4.48	1.86	3.12	6.98	2.85
	3	10.67	4.50	8.00	11.13	10.67
	6	2.13	3.97	2.26	2.27	2.22
	9	4.53	3.26	4.58	4.45	4.18

The recorded pH values ranged from 6.45 to 8.36 across all treatments and periods. While pH is a critical factor in metal solubility, the stability observed within this circumneutral range suggests that pH-induced acid leaching was not the primary driver for metal mobility in this specific experimental setup (U.S. Epa 2007; Sposito 2008).

Conductivity values showed significant variation, with peaks observed in reactors containing centrifuged sludge (CC and UBSN), reaching up to 1482 µS/cm. Although values above 100 µS/cm can be associated with anthropogenic impact in surface waters (CETESB 2018a), in the context of this work, these levels may reflect the natural release of soluble ions from the WTPS sludge matrix, such as residual salts from the coagulation process.

The variation in the volume of the percolate is expected and indicates good representativeness of the system, since first the empty spaces are filled by water and, subsequently, the excess volume begins to move under the action of gravity, characterizing percolation (Hillel 1998).

Table 6 shows the maximum metal concentrations identified in the analyses during the rainy and dry seasons. Considering the precautionary principle, environmental control of potential contaminants should consider the highest concentrations observed in leachate (IPT 2014). It is noteworthy that Co, Cr, and Cd are not depicted in the table because their concentrations remained consistently below the instrument’s limit of detection (LOD = 0.00020 mg/L for Co, 0.00020 mg/L for Cr, and 0.00005 mg/L for Cd.

**Table 6:** Concentrations (mg/L) of trace metals and major elements in percolates collected from experimental reactors during rainy and dry seasonal periods

Metal	Reactor	Concentration [mg/L]		Metal	Reactor	Concentration [mg/L]	
		Rainy months	Dry months			Rainy months	Dry months
Al	CS	0.06	0.07	Ba	CS	0.03	0.04
	CC	0.14	0.06		CC	0.13	0.75
	CSN	0.13	0.20		CSN	4.07	2.00
	UBC	1.18	1.37		UBC	0.09	0.17
	UBSN	0.29	0.33		UBSN	2.97	0.90
Cu	CS	0.13	0.00	Fe	CS	0.00	0.06
	CC	0.04	0.01		CC	2.74	20.52
	CSN	0.01	0.01		CSN	0.09	0.06
	UBC	0.01	0.00		UBC	0.77	0.87
	UBSN	0.05	0.00		UBSN	0.20	0.19
Mg	CS	0.52	1.16	Mn	CS	0.18	0.02
	CC	4.15	6.86		CC	8.26	40.05
	CSN	56.25	12.47		CSN	90.16	48.31
	UBC	1.70	2.96		UBC	2.57	0.32
	UBSN	43.34	13.69		UBSN	69.30	19.82
Ni	CS	0.00	0.02	Pb	CS	0.13	0.05
	CC	0.01	0.01		CC	0.15	0.03
	CSN	0.01	0.03		CSN	0.15	0.05
	UBC	0.00	0.00		UBC	0.16	0.02
	UBSN	0.00	0.00		UBSN	0.16	0.02
Zn	CS	0.00	0.02				
	CC	0.02	0.02				
	CSN	0.07	0.08				
	UBC	0.00	0.00				
	UBSN	0.05	0.00				

CS = Soil; CC = Centrifuged Sludge; CSN = Naturally Dried Sludge; UBC = Beneficial Use—Centrifuged Sludge; UBSN = Beneficial use—Naturally Dried Sludge

Despite WTPS classification as non-hazardous waste (ABNT 2024a, b, c) and WTPS beneficial application being legally permitted (CONSEMA 2022), the results showed the potential for contamination by toxic metals like Al, Ba, Cu, Ni, Pb, Mg, Mn, and Zn. Otherwise, all these metals (except Mn) were found in soil samples (CS), which indicates that the potential contamination is not only from the WTPS.

In general, the highest concentrations of metals were found in the analyses of rainy months, which indicates that surface runoff initially occurred (Dykyjová et al. 1983) of the elements and, subsequently, the absorption of the metals by soil (Almeida et al. 2025). Besides, high volumes of rainfall can intensify metal concentrations and nutrient availability in water (Xian et al. 2024).

Mn and Mg stood out as the metals with the highest concentrations in the WTPS, which can be seen as originating from the WTPS analyzed. Mn occurs naturally in aquatic surfaces (WHO 2011); however, it is required in small quantities because the margin between their deficiency and toxicity is generally narrow (Antonkiewicz et al. 2025). High Mg values can be justified because this metal is abundant in São Paulo State soils (CETESB 2018b) or may indicate contamination by irregular discharges of organic matter (Kanmani & Gandhimathi 2013).

Except for Fe and Mn, all metals found in the WTPS were also found in the soil, but in lower concentrations. Furthermore, it is possible to infer that the concentrations of most metals found in the WTPS and soil decreased in the leachate analyses of the beneficial simulations, indicating a positive impact of the mixture.

Table 7 shows the highest concentrations of metals found in the leachate and the limits for these concentrations established by environmental legislation. Highlighted are those metals that exceeded at least one of these concentrations.

**Table 7:** Maximum metals concentration and theirs respectively limits in the regulamentation

Metal	Brazil (CONAMA, 396/2008) [mg/L]	European Union (EU 2020/2184) [mg/L]	United States (MCL,USEPA, 2025) [mg/L]	Maximum value founded [mg/L]				
				CS	CC	CSN	UBC	UBSN
Al	0.050	0.200	N/A	**0.070**	**0.140**	**0.200**	**1.370**	**0.330**
Ba	0.020	N/A	2.000	**0.040**	**0.750**	**4.070**	**0.170**	**2.970**
Cd	0.005	0.005	0.005	0.000	0.000	0.000	0.000	0.000
Pb	0.001	0.005	0.010	**0.130**	**0.150**	**0.150**	**0.160**	**0.160**
Cu	0.050	2.000	1.300	**0.130**	0.040	0.010	0.010	0.050
Cr 6 +	0.100*	0.025	0.100	0.000	0.000	0.000	0.000	0.000
Cr 3 +	1,000*	0.025	0.100	0.000	0.000	0.000	0.000	0.000
Co	0.001	N/A	N/A	0.060	0.000	0.000	0.000	0.000
Fe	0.100	N/A	N/A	0.049	**20.520**	0.090	**0.870**	**0.200**
Mg	N/A	N/A	N/A	1.160	6.860	56.250	2.960	43.340
Mn	0.025	0.050	N/A	**0.180**	**40.050**	**90.160**	**2.570**	**69.300**
Ni	0.001	0.020	N/A	**0.020**	**0.010**	**0.150**	0.000	0.000
Zn	0.010	N/S	N/A	0.020	**0.020**	**0.080**	0.000	**0.050**

*Considered the value in CONAMA 430 (BRASIL, 2011)

The data in Table 7 show that legislation converges only on the maximum limit for *Cd*. For the other metals evaluated, Brazilian legislation is the most restrictive. Furthermore, it is observed that Brazil sets limits for the concentration of *Al* and *Fe* in liquid samples, metals potentially toxic to freshwater organisms such as microalgae (Bernegossi et al. 2022), but does not set this value for solid samples (BRASIL, 2009).

Mn was the metal found in the highest concentrations in the reactors containing sludge. This metal, although essential to chlorophytes, can be toxic in high concentrations, causing reduced algal growth rates. There are few studies discussing the toxicity of Mn to *Raphidocelis subcapitata*; however, the available data (~1.0 mg/L) indicate effects at concentrations much lower than those observed in the present study. Even so, the values found of Mn are toxic even to humans (WHO 2011; U.S. Epa 2023; CETESB 2023).

Some studies indicate no effect on algae growth rate at Ba concentrations below 1.15 mg/l (ECHA, 2024)—limit exceeded in reactors containing naturally dried sludge. Zn, Pb, and Ni can inhibit cell growth at concentrations of about 15.0 mg/l, 0.16 mg/l, and 0.5 mg/l, respectively (Khellaf & Zerdaoui 2009; Alho et al. 2019; Reis et al. 2024)—concentrations that were not observed in the reactors. The Cu concentrations observed in reactors CS, CSN, and UBSN have the potential to retard cell growth and reduce algal populations (concentration > 0.03 mg/l) (Machado & Soares 2024a, b).

Finally, comparing WTPS with sewage sludge reveals distinct element partitioning. While sewage sludge exhibits higher organic nutrient loads (N and P) and even microplastics (Hassan et al. 2023), both matrixes share trace element dynamics that govern their ecotoxicological risks and resource recovery potential (Souza et al. 2024). Depending on concentration and mobility, these elements act as either micronutrients or environmental constraints (Antonkiewicz et al. 2025; Souza et al. 2024).

### Ecotoxicological analysis

The value of inhibitory concentration (IC) for the microalgae to K_2_Cr_2_O_7_ was within the previously established sensitivity range, indicating good physiological conditions for the microalgae. Table 8 shows the results of toxicity tests with the microalgae *R. subcapitata* in terms of cell density, per month of leachate collection, and per reactor.

**Table 8:** Mean values of the cellular density of *R. subcapitata* in toxicity tests with reactors samples

Algal count (*10^4^)					
Month	Reactors	Mean ± SD	Month	Reactors	Mean ± SD
12	Control	58 ± 1	**6**	Control	84 ± 3
	CC	11 ± 2		CC	10 ± 2
	CSN	13 ± 1		CSN	9 ± 2
	CS	44 ± 3		CS	40 ± 3
	UBC	50 ± 3		UBC	37 ± 3
	UBSN	19 ± 2		UBSN	7 ± 1
3	Control	63 ± 3	**9**	Control	73 ± 4
	CC	7 ± 1		CC	4 ± 1
	CSN	14 ± 2		CSN	13 ± 2
	CS	54 ± 2		CS	61 ± 5
	UBC	61 ± 6		UBC	60 ± 6
	UBSN	12 ± 2		UBSN	9 ± 2

Based on the average values of algal density, it is noted that the control reactor containing centrifuged sludge (CC) was the most toxic, followed by the reactors containing naturally dried sludge (CSN and UBSN). In general, the toxicity of leachate from WTPS or soils was related to a greater variety of chemical elements present (Rodgher et al. 2023). For these samples, Al, Ba, Cu, Mg, Mn, Pb and Zn were quantified.

Furthermore, Potentially Toxic Elements (PTEs) (e.g., Cu, Ni, Zn and Pb) (Panigua-López et al. 2023) were found in leachate analyses of both the sludge and the soil. Considering that algal density decreased in all samples studied, it is not possible to confirm that the toxicity to *R. subcapitata* was due exclusively to the leachate studied. On the other hand, it is noted that the average algal density values increased when adding soil to the centrifuged sludge (UBC), indicating a positive effect of the mixture.

CC showed higher toxicity than UBC. Although both refer to the same drying process, this difference may be explained by the soil characteristics: Clay soils act as an efficient geochemical barrier; their high specific surface area and elevated Cation Exchange Capacity (CEC), coupled with the presence of Fe and Al oxides, promote the immobilization of metallic ions through adsorption, ion exchange, and surface complexation (Bradl 2004; Sposito 2008). Comparing the leachate analysis of CC and UBC it is possible to observe that this process may have occurred. The concentrations of Cu, Fe, Mg, Mn, Ni and Zn were higher in CC than UBC. For the metals Cu, Ni and Zn the difference between both reactors is up to 100%. This reduction indicates that the soil addition in the UBC reactor was able to retain these metals within its mineral structure, preventing their release into the aqueous phase and, consequently, reducing the bioavailability of these elements to the microalgae *R. subcapitata.*

The comparison between the reactors containing only sludge revealed higher toxicity in CC compared to CSN. Given that CSN underwent an additional drying process—reducing the moisture content from 82% (CC) to 50% (CSN)—this reduction in toxicity suggests that the further removal of water and the possible volatilization of labile compounds during drying may have mitigated the inhibitory effects on microalgae. Although specific volatile substances were not quantified, these results align with findings that different sludge treatment intensities significantly alter their chemical properties and subsequent ecotoxicological profile (Ospanov et al. 2024; Azeddine et al. 2022).

Furthermore, the sludge drying process constitutes an important factor that influences the concentration of metals detected, as the reduction in moisture content proportionally increases the solids fraction (Silva et al. 2025). This condition is likely to have contributed to the higher toxicity observed in both beneficial scenarios (UBC and UBSN), given that naturally dried sludge exhibits a higher solids content compared to centrifuged sludge (~36%). Consequently, the difference in toxicity between these reactors ranged from 15 to 32%.

To evaluate the influence of WTPS drying methods on algal density, data normality was first assessed using the Shapiro–Wilk test. Due to the non-normal distribution observed in some groups (*p* < 0.05), a non-parametric approach was adopted. The Kruskal–Wallis test revealed a statistically significant difference between the reactors (*χ*^2^ = 120; df = 5; *p* < 0.001). The calculated effect size, expressed by Epsilon-squared (ε^2^ = 0.839), indicates a very strong effect, suggesting that approximately 83.9% of the observed variation in algal density is directly attributed to the reactor composition and drying processes.

**Fig. 5:**
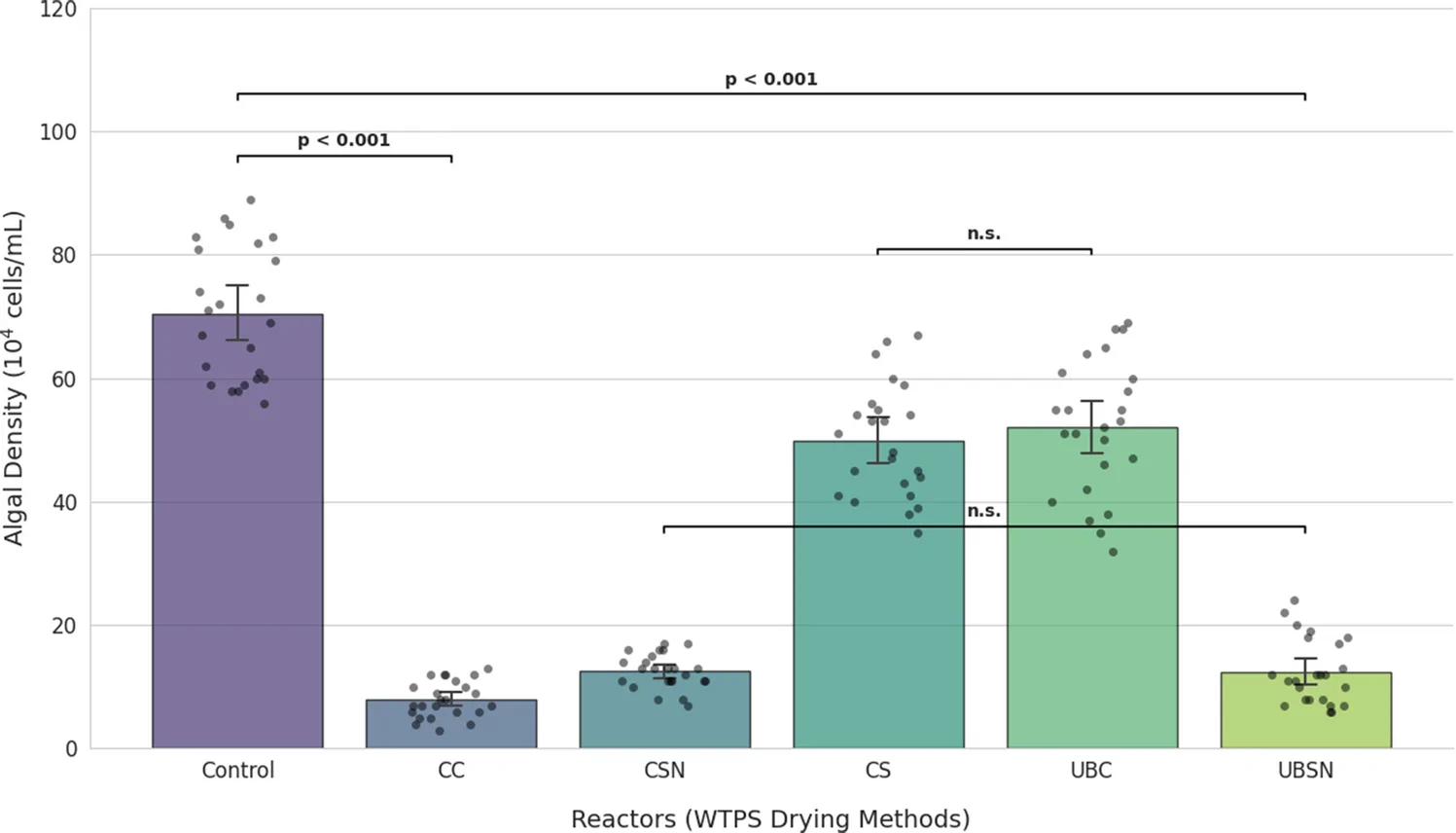
Algal density observed in the different reactors according to the WTPS sludge drying methods. Bars represent mean values, and error bars indicate standard deviation. Individual data points (diamonds) show the distribution of all replicates (*N* = 24). Statistical significance was confirmed by the Kruskal–Wallis test *(χ*^*2*^ = 120, *p* < 0.001), with a strong effect size (*ε*^2^ = 0.839). Brackets indicate comparisons with *p* < 0.001 or non-significance (n.s., *p* > 0.05) based on the DSCF post-hoc test

These differences are visually summarized in Fig. 5, where all experimental treatments showed a significant reduction in algal counts compared to the Control (*p* < 0.001). Post-hoc analysis using the Dwass–Steel–Critchlow–Fligner (DSCF) test further detailed these variations, confirming statistical parity between treatments using naturally dried sludge (CSN and UBSN; *p* = 0.973) and between the CS and UBC combination (*p* = 0.972). All other pair comparisons showed significant differences, highlighting the distinct efficiency levels of each drying method.

This finding justifies the current concern about the environmental risks associated with WTPS beneficial application, since, unlike organic compounds, metals like these found in WTPS composition are not biodegradable (Siegel 2002). However, it is crucial to consider the potential migration routes of the pollutant. To be assimilated, metals must be mobile, transport to the organism, and be bioavailable (Bradl 2005). Thus, it is evident that metal analysis is an essential factor for the safe and sustainable use of WTPS, together with all the environmental conditions to which the WTPS will be exposed.

**Fig. 6:**
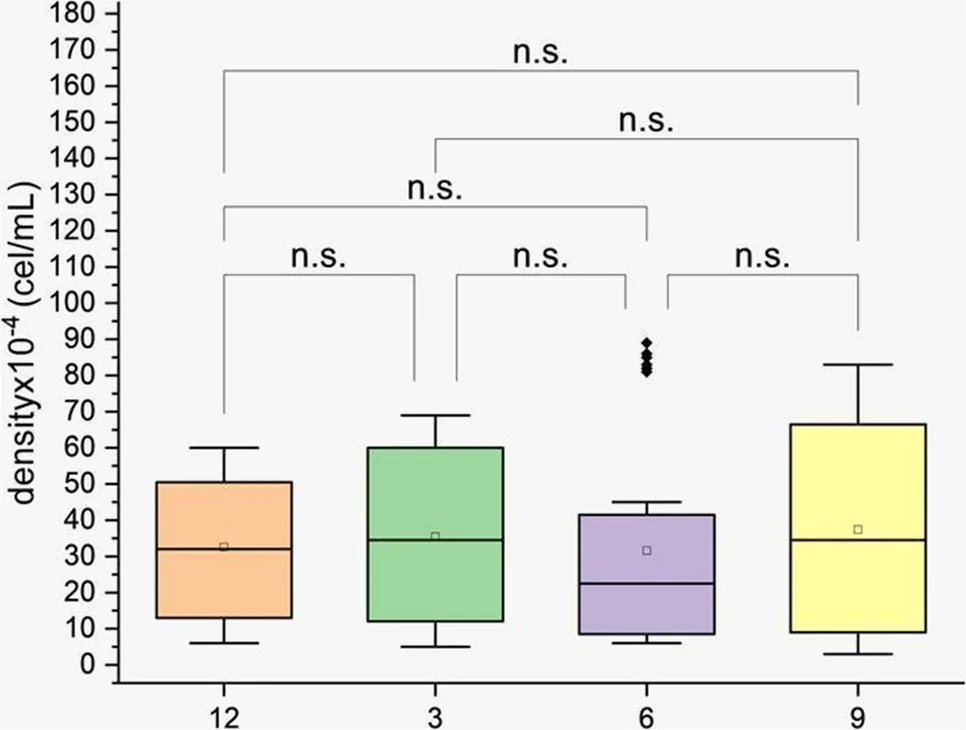
Seasonal variation of algal density across the simulated periods. The data points represent the mean algal density for each month. The non-significant *p*-value (*p* = 0.103, Friedman test) indicates that seasonality did not exert a decisive influence on the system’s biological performance. Annotations “ns” (not significant) indicate *p* > 0.05 for all pairwise comparisons

Regarding seasonal variations, the Shapiro–Wilk test indicated a non-normal distribution for algal density data across all sampling months (*p* < 0.05). Consequently, the non-parametric Friedman test was employed to compare the rainy and dry seasons. The analysis showed no statistically significant differences in algal density between the periods (*χ*^2^ = 6.19; df = 3; *p* = 0.103). This stability in biological response suggests that, despite potential chemical variations in sludge composition throughout the year, the impact on microalgae remained consistent across the simulated seasonal scenarios (Fig. 6).

Prior study on WTPS toxicity has reported marked differences between sludge samples collected during rainy and dry seasons. Variations were mainly observed in turbidity, solids content, and Al and Fe concentrations; nevertheless, these parameters did not show a consistent relationship with toxicity (Sotero-Santos et al. 2005).

Although our results indicate a stable biological response of the system under the different seasonal scenarios tested, Messa et al. (2025) reported that seasonal variability can influence sludge composition characteristics due to the input loads added to maintain the quality of the treated water, generating consequences in the subsequent stages of sludge treatment (Silva et al. 2025).

## Conclusion

The results of this study demonstrate that the methodologies applied were effective in evaluating the toxicological profile of the *in natura* percolate—the actual effluent that interacts with the environment. This approach provides a more realistic assessment of WTPS reuse in geotechnical works.

The findings suggest that the WTPS drying method plays a decisive role in the biological response of *R. subcapitata*. While the centrifuged sludge percolate (CC) exhibited the highest growth inhibition, its incorporation into the soil matrix (CS) significantly reduced toxicity, reaching the lowest inhibitory levels observed. This indicates that the soil-sludge interaction may mitigate the bioavailability of contaminants. Conversely, seasonal variations (dry vs. rainy) did not significantly influence the toxicological outcomes.

Although the presence of metals such as Mn, Mg, Zn, Pb, Ba, and Cu was identified in both the soil and WTPS, the individual contribution of each matrix to the overall toxicity remains to be further elucidated.

Despite the insights provided, this study was constrained by logistical and financial requirements associated with specialized outsourced laboratory analyses, which limited the assessment to four seasonal sampling events. While these samples captured the representativeness of the dry and rainy months, further studies with increased sampling frequency are recommended to evaluate temporal toxicological variability.

Regarding future research, it is recommended to quantify residual organic coagulants, polyelectrolytes, and emerging organic micro-contaminants to assess non-metal toxic drivers in WTPS percolates. Furthermore, expanding the ecotoxicological battery to other trophic levels—specifically incorporating benthic organisms (e.g., *Chironomus*) to evaluate sediment-sludge interactions—is essential. Finally, utilizing pristine control soil (collected from an area with no anthropogenic impact) and testing neat WTPS independently would allow for a clearer isolation of the synergistic effects occurring within the soil-sludge matrix.

## Supplementary Information

Below is the link to the electronic supplementary material.

ESM 1 (DOCX 150 KB)

## Data Availability

After publication, the data will be available on demand to authors.
