# Supplementary material for: Toxicity of percolates from geotechnical applications with water treatment sludge incorporation
Source: Environ Sci Pollut Res Int. 2026 Oct 21;33(38):17618–32. doi: 10.1007/s11356-026-38303-9 (PMC13740104; doi:10.1007/s11356-026-38303-9)
Supplement: Supplementary file 1 [file 11356_2026_38303_MOESM1_ESM.docx]

**Sensitivity Control Cards**

Sensitivity of the algae *Raphidocelis subcapitata* to the reference substance Potassium Dichromate - K_2_Cr_2_O_7_(CAS 7778-50-9 - Synth Brand - Lot 259372 - Expiry Date 10/02/2028).

## **Test Month 3**

Test Period: 23/08/2021 to 11/12/2023

Last Test Result (11/12/2023): EC(I)_50_;72h=1.37 mg K_2_Cr_2_O_7_/L

- Mean: 1.57 mg/L
- Lower Control Limit (LCL): 0.82 mg/L
- Upper Control Limit (UCL): 2.33 mg/L

## **Test Month 6**

Test Period: 23/08/2021 to 18/03/2024

Last Test Result (18/03/2024): EC(I)_50_;72h = 1.07 mg K_2_Cr_2_O_7_/L

- Mean: 1.57 mg/L
- Lower Control Limit (LCL): 0.82 mg/L
- Upper Control Limit (UCL): 2.33 mg/L

## **Test Month 9**

Test Period: 23/08/2021 to 16/07/2024

Last Test Result (16/07/2024): EC(I)_50_;72h = 1.75 mg K_2_Cr_2_O_7_/L

- Mean: 1.57 mg/L
- Lower Control Limit (LCL): 0.82 mg/L
- Upper Control Limit (UCL): 2.33 mg/L

## **Test Month 12**

Test Period: 23/08/2021 to 03/10/2023

Last Test Result (16/07/2024): EC(I)_50_;72h = 1.95 mg K_2_Cr_2_O_7_/L

- Mean: 1.57 mg/L
- Lower Control Limit (LCL): 0.82 mg/L
- Upper Control Limit (UCL): 2.33 mg/L

**Graphics:**
